# Determining the pre-grazing sward height of Kikuyu grass (*Cenchrus clandestinus* - Hochst. ex Chiov.) for optimizing nutrient intake rate of dairy heifers

**DOI:** 10.1371/journal.pone.0269716

**Published:** 2022-07-08

**Authors:** Alejandra Marín Gómez, Emilio A. Laca, Tiago Celso Baldissera, Cassiano Eduardo Pinto, Fábio Cervo Garagorry, Angel S. Zubieta, Carolina Bremm, Jerôme Bindelle, Paulo César de Faccio Carvalho

**Affiliations:** 1 Grazing Ecology Research Group, Federal University of Rio Grande do Sul, Porto Alegre, Brazil; 2 Facultad de Ciencias Agrarias, Departamento de Producción Animal, Universidad Nacional de Colombia, Medellín, Colombia; 3 Department of Plant Sciences, University of California, Davis, California, United States of America; 4 Empresa de Pesquisa Agropecuária e Extensão Rural de Santa Catarina (Epagri), Lages, Santa Catarina, Brazil; 5 Precision Livestock and Nutrition Unit, AgricultureIsLife, TERRA Teaching and Research Centre, Gembloux Agro-Bio Tech, Liège University, Gembloux, Belgium; Tokat Gaziosmanpasa Universitesi, TURKEY

## Abstract

Understanding the grazing process and animal response to sward structures (e.g., sward height) is key to setting targets for efficient grazing management. We hypothesized that the short-term intake rate (STIR) of dry matter (DM) and digestible organic matter (OM) by dairy heifers is maximized with Kikuyu grass (*Cenchrus clandestinus—*Hochst. ex Chiov.) of intermediate sward heights. The treatments consisted of five pre-grazing sward heights (10, 15, 20, 25, and 30 cm) randomly assigned to two of ten paddocks. The experimental design included two measurements of each paddock at different periods and times of day. Three Holstein heifers (440 ± 42 kg body weight) were used to determine the STIR, which was estimated using the double-weighing technique with correction for insensible weight losses. The bite mass (BM), bite rate (BR), sward structural characteristics, and nutritional value of herbage samples were assessed. The data were analyzed using mixed models with a factorial arrangement of five sward heights, two times of day, and two evaluation periods. The sward height of Kikuyu grass that maximized both STIRs was approximately 20 cm. The STIR of the DM was 30% and 15% lower than the maximum in the shortest and tallest swards tested, respectively. In swards shorter than 20 cm, the STIR was lower because the BM decreased with sward height, whereas in those greater than 20 cm, the lower BM and STIR of DM was explained by a decrease in bulk density and bite volume. The top stratum was composed mainly of highly digestible leaf blades with similar nutrient content across sward heights; therefore the STIR of digestible OM was also maximized at 20 cm. Hence, the optimal pre-grazing sward height of Kikuyu grass should be managed at 20 cm under rotational stocking systems to maximize nutrient intake rate of dairy heifers.

## Introduction

The intake of dry matter (DM) and digestible nutrients is an essential driver of livestock productivity [1]. In dairy production systems where cows are stall-fed, the quantity of nutrients offered is highly controlled and intake per unit of feeding time is greater and more efficient than pasture-based systems because feeds are nutrient and energy-dense [2,3]. In pasture-based dairy systems, forage is spread over large areas, and the rate of food intake is determined by bite mass (BM) and the time required to find, gather, chew, and swallow each bite [4,5]. The instantaneous intake rate during grazing is the product of the BM and bite rate (BR) [6]. On a daily scale, intake rate is also affected by the time required for rumination and digestion, social interactions, rest, and milking [7–9]. In this context, intake per unit of feeding time, hereafter referred to as short-term herbage intake rate (STIR), generally limits daily intake, and increases in STIR translate into greater productivity [10]. Therefore, if grazed herbage is the main source of nutrients for dairy cows, it is pivotal that animals have continuous access to pastures with structures that allow for the maximum STIR.

Sward structure plays an important role in the grazing process and determines the STIR [11–13]. Among the various sward structural characteristics (e.g., sward height, bulk density, herbage mass, and leaf: stem ratio), sward height has been identified as the main factor determining bite mass (BM) and STIR [12,14,15]. Therefore, to sustain high DM and nutrient intake rates, optimal sward height should be a grazing management target [10]. *Rotatinuous* stocking was proposed as a grazing concept to optimize and sustain STIR by maintaining swards at an optimal height for animal grazing that differs among forage species [10,14,16–18].

A high herbage intake rate combined with a high quality diet is key in pasture-based dairy production systems, especially in the tropics [19,20], where low levels of performance are partly attributed to the intrinsic lower quality of C4 compared to C3 grasses [21], among other things. Kikuyu grass (*Cenchrus clandestinus—*Hochst. ex Chiov) is a subtropical species from East Africa that is widely used in the dairy systems of some regions, including Africa, Latin America, Australia, and New Zealand [22–24]. As optimal heights differ among forage species, farmers need to know the targets for their particular species. Hence, we aimed to determine the Kikuyu grass sward height that maximizes DM and digestible OM intake rate for dairy heifers and identify the mechanisms that explain the existence of such optimal height. We tested the hypotheses that 1) the short-term intake rate (STIR) of DM by dairy heifers is maximized at intermediate sward heights of the Kikuyu grass, and that 2) the maximum STIR of digestible OM occurs at the same sward height as that of DM. This study contributes to a better understanding of the grazing process and how animals respond to the sward height of subtropical grass species such as Kikuyu grass, and it provides a promising pre-grazing management sward height target for optimizing the nutrient intake rate of dairy heifers under rotational stocking.

## Materials and methods

### Ethics statement

This study was conducted in accordance with the recommendations of the Ethical Review Committee on the Use of Animals at the Federal University of Rio Grande do Sul, Brazil (approved project no. 33970) for separate experiments using the same non-invasive ingestive behavior protocol, because at the time of this study in 2017, the Animal Ethics Committee at the Santa Catarina Agricultural Research and Rural Extension Company was still being formed. The experimental animals were used in accordance with the Guidelines for the Care and Use of Agricultural Animals in Agricultural Research and Teaching for Scientific Purposes [25].

### Experimental site

The study was conducted at the Agricultural Research and Rural Extension Company of Santa Catarina (EPAGRI), municipality of Lages, S. C, Brazil (27°47ʹ10.5ʺ S, 50°18ʹ20.5ʺ W, 937 m.a.s.l.). According to the Köppen climate classification, the regional climate is Cfb-type. The mean annual temperature is 16.8°C and the mean annual average precipitation is 1460 mm [26]. According to the USDA Soil Taxonomy [27], the soil of the experimental area was classified as humudept (with an umbric epipedon) [28].

The experiment was performed in a 5000 m^2^ permanent pasture of Kikuyu grass (*Cenchrus clandestinus—*Hochst. ex Chiov), established in the early 1990s that has been grazed by dairy and beef cattle since inception. The entire area was mowed to 5 cm of height (all cuttings were removed) and divided into ten paddocks of 500 ± 5 m^2^ on January 15, 2017. The entire pasture received one application of 250 kg/ha of fertilizer (N-P-K, 9–33–12) and 135 kg/ha of urea on January 26, 2017 (first evaluation period). On March 22, 2017, 67.5 kg/ha of urea was applied (second evaluation period). Due to frost events and low temperatures in winter and possibly spring, Kikuyu dies at the end of autumn and regrows at the end of spring [29]; therefore, the data collection in this study lasted from February 28 to April 15, 2017.

### Treatments and experimental design

Treatments consisted of five pre-grazing sward heights (10, 15, 20, 25, and 30 cm) of Kikuyu grass. Each treatment was randomly assigned to two out of ten paddocks. One paddock of each sward height was randomly assigned to a morning grazing session, and the other was assigned to an afternoon session. This resulted in a factorial of sward height and time of day, in which each treatment was applied to one experimental unit. Each paddock was grazed and the corresponding treatment was observed once during the first experimental period. Subsequently, each paddock was mowed to a height equal to half of its nominal height treatment and allowed to grow back until it reached the same target height used in the first period. The time-of-day factor was reversed for each paddock, and all paddocks were grazed for a second time during the second experimental period. Due to time constraints, only two paddocks were grazed and observed per day, once in the morning and once in the afternoon. The order in which the treatments were observed was randomized for each period. This resulted in 20 short-term grazing sessions.

The experimental design included two measurements of each physical paddock, albeit in different periods and times of the day. Therefore, data were analyzed with mixed models, including a random effect for the paddock, to account for the possible intraclass correlation of errors.

### Animal measurements and grazing sessions

Three Holstein Friesian heifers with an average body weight of 440 ± 42 kg and 22 ± 2 months of age were used to determine the STIR. Short-term intake rates were assessed by weighing the animals before and after grazing and correcting for insensible weight losses (water evaporation, respiratory losses, carbon dioxide, and methane) as described in the double-weighing technique [30]. Heifers were familiarized with the experimental protocol one month before beginning the grazing trial and maintained in an adjacent area similar to the experimental paddocks with free access to Kikuyu grass. The same group of three dairy heifers grazed each paddock once in the morning and in the afternoon during each experimental period.

The animals were allowed to graze for 45 min during peak grazing times at 7:30 and 16:00 h (the first and last grazing meals, respectively [31]). The grazing session lasted 45 ± 5 min, which was considered the minimum time to detect body weight fluctuations [9]. The paddock area (500 ± 5 m^2^) was calculated to result in a less than a 10% change in average sward height during the grazing session, ensuring that the same sward structure at the bite level was available for the animals to graze over the entire course of each grazing session.

Before each grazing session, the three heifers were fitted with a feces and urine collection bag and an Institute of Grassland and Environmental Research (IGER) Behaviour Recorder (Ultra Sound Advice, London, UK) [32], which records the effective eating time (ET, the length of time that an animal spends eating during grazing) and number of grazing (biting and non-biting) jaw movements. The animals were weighed (W1) on an electronic scale (10 g precision), and turned into the appropriate paddock. The precise pre-grazing time (t1, min: s) was recorded with a timepiece synchronized to the IGER Behavior Recorder clock. After each grazing session, the animals were returned to the handling area and weighed again (W2). The precise time was recorded (t2) and the IGER Behaviour Recorder was removed. Heifers were weighed (W3) and the time was recorded (t3); they were left in the handling area without feed or water for 45 ± 5 min to measure insensible weight losses. The heifers were weighed again and the precise time (t4) was recorded. During grazing sessions, feces and urine were collected totally without leakage. The animals were not fasted at any time to avoid any alteration in their ingestive behavior [33,34] and diet selection [35]. Finally, all equipment was removed, and the animals were released in an adjacent area with free access to Kikuyu grass. The ET was determined by analyzing the grazing behavior recording using the software “Graze” (Ultra Sound Advice, London, UK) [36]. Thus, the STIR on a fresh-matter basis was calculated as follows:

$$STIR=\left\{\left[\frac{\left(W_{2-}W_{1}\right)}{\left(t_{2-}t_{1}\right)}\right]+\left[\frac{\left(W_{3-}W_{4}\right)}{\left(t_{4-}t_{3}\right)}\right]\right\}\left[\frac{\left(t_{2-}t_{1}\right)}{ET}\right]$$ (1)

where W1 and W2 are pre- and post-grazing animal weight (kg), respectively; t1 and t2 pre- and post-grazing time (min), respectively; W3 and W4 are pre- and post-insensible weight losses (kg), respectively; t3 and t4 are the pre- and post-insensible weight losses time (min), respectively; and ET is effective eating time (min). ET was calculated as the total session time excluding intervals of jaw inactivity greater than 3 s [31]. The STIR of DM for each treatment was calculated as the STIR of fresh matter multiplied by forage DM content, which was estimated based on the hand-plucked herbage samples (as described below).

The STIR of digestible OM (g/min) was calculated as the product of the STIR of DM and *in vitro* OM digestibility (g/kg) obtained from grazing herbage samples simulated by hand-plucking. The bite mass (BM, g DM) was calculated as the product of the STIR and ET divided by the total number of bites. The bite rate (BR, bites per min) was determined for each animal and each grazing session by dividing the total number of bites by effective eating time.

### Sward measurements

Sward height was measured at 150 haphazardly selected points per paddock, distributed over the entire paddock before and after each grazing session using a Hill Farming Research Organization type sward stick [37]. Pre-grazing herbage mass was assessed using three random herbage samples clipped at ground level using a metallic quadrat of 0.25 m^2^. Each fresh herbage sample was immediately separated into leaf laminae, stems + sheaths, and dead material, and dried in a forced-air oven at 55°C for 72 h. The total herbage mass (kg DM/ha) was the sum of the masses of all components. Bulk density (g DM/m^3^) of the individual sward components was calculated as the corresponding clipped dry mass divided by sward volume clipped, which was the product of the quadrat area (0.5 × 0.5 m) and average sward height. Four representative herbage samples (~200 g fresh weight) were hand-plucked [38], two at the beginning and two at the end of each grazing session, mimicking the closely observed grazing behavior of the heifers.

### Herbage chemical analysis

Hand-plucked herbage samples were analyzed in duplicate for dry matter (DM; method 930.04; [39]), ash (method 930.05; [39]), and neutral detergent fiber (NDF), and acid detergent fiber (ADF) [40] by using an ANKOM 200 fiber analyzer without heat-stable alpha-amylase. NDF and ADF were expressed including residual ash. Samples were also characterized for N content by the Kjeldahl digestion (method 984.13; [39]). Crude protein (CP) was calculated as N concentration × 6.25. A two-stage [41] technique (incubation with rumen fluid followed by acid-pepsin digestion) was used to estimate the *in vitro* OM digestibility (IVOMD).

### Statistical analyses

Statistical analyses were performed using R software version R 3.5.3 [42]. The relationship between ingestive behavior data (STIR of DM, BM, BR, and STIR of digestible OM) and sward height (SH) was analyzed as described by [17], which consists of fitting a double linear model or broken line model, as follows: y = *f*{*p*+*a*1(*SH—v*),*p* + *a*2(*SH—v*)}, where y is the STIR of DM, BM, BR, and STIR of digestible OM, f is the min (STIR of DM, BM, and STIR of digestible OM) or max (for BR) function; v and p are the coordinates of the point where lines cross; SH is the observed average value of sward height; and a1 and a2 are the slopes of the component lines. Before assessing treatment effects, values were corrected by subtracting the animal effects as follows: corrected Y = (original observation − animal average) + overall average. Ingestive behavior models were fitted to the data by deviance minimization using the optim{stats} function of the stats package in the R 3.5.1 [42]. We also used a quadratic equation to model the relationship between ingestive behavior data and sward height, and compared it to the broken line model using Akaike’s information criterion (AIC).

Nutrient content and sward variables were analyzed using linear mixed-effects models with the lmer function of the lme4 package [43]. Treatment, time of day, period and their two-way interactions were the fixed effects and paddock was the random effect in the model: y ~ (treatment + time of day + period) ^2 + (1 | paddock). Significance was declared at P < 0.05 and tendencies at 0.05 < P ≤ 0.10. Residuals of the analyses were checked for normality using the Shapiro-Wilk normality test with the shapiro. test function in R [42].

## Results

### Sward structure and nutritional value

The pre-grazing sward heights obtained were close to the nominal treatment heights, and the differences between the pre-and post-grazing sward heights did not exceed the predetermined maximum of 10% of the initial height (Table 1). There were interactions among the treatments with the time of day and period of evaluation for the actual pre-and post-grazing sward heights (P ≤ 0.001, S1 Text), showing slight reductions in 20 and 25 cm sward heights from the morning evaluation and Period 1 (Table 1).

**Table 1:** Pre- and post-grazing sward heights of Kikuyu grass (*Cenchrus clandestinus—*Hochst. ex Chiov) at two times of day in two evaluation periods.

	Treatment					
	10	15	20	25	30	
	Pre-grazing Sward height (cm)					SEM
Time of day (AM)	10.0	15.9	19.7	23.7	32.3	0.34
Time of day (PM)	9.6	14.4	20.5	24.8	30.3	0.34
Period 1	10.2	15.7	19.6	24.5	30.8	0.34
Period 2	9.4	14.4	20.6	24.0	31.8	0.34
	Post-grazing Sward height (cm)					SEM
Time of day (AM)	9.5	15.1	17.8	22.1	30.0	0.56
Time of day (PM)	9.1	13.7	19.4	23.2	28.6	0.56
Period 1	9.4	15.2	17.8	22.6	29.5	0.56
Period 2	9.2	13.6	19.3	22.7	29.1	0.56

The times of the day AM and PM correspond to the morning and afternoon assessment respectively. SEM: Standard error of the mean.

Overall, there were interactions between treatments and the time of day and period for all the sward structure variables (S2 Text). No significant interactions were found between the time of day and period for any sward variable (S2 Text).

**Fig 1:**
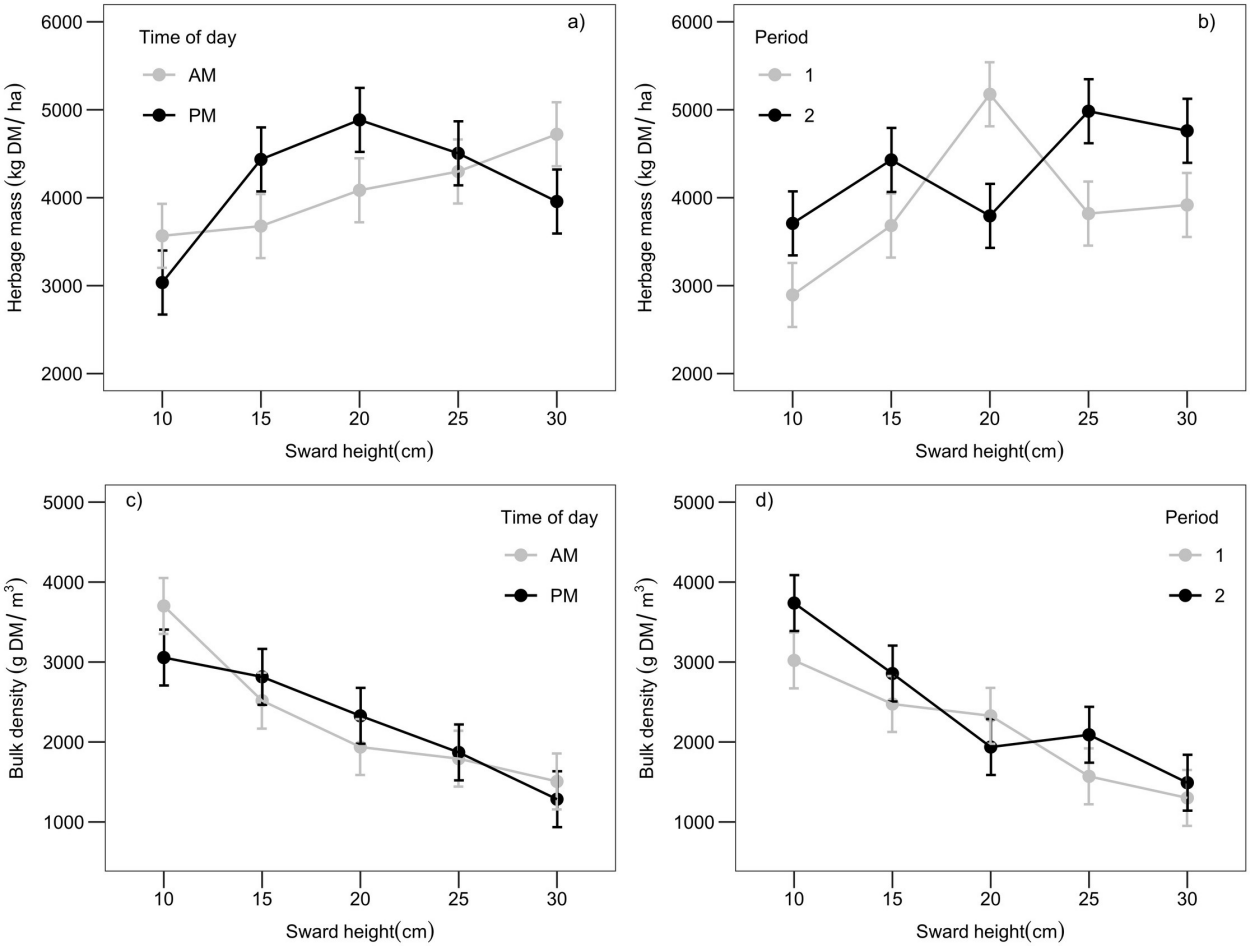
Interactions between sward heights of Kikuyu grass and time of day and period of evaluation. Interactions between sward heights (10, 15, 20, 25, and 30 cm) of Kikuyu grass (*Cenchrus clandestinus—*Hochst. ex Chiov) and time of day (morning, AM, and afternoon, PM) on herbage mass (a) and bulk density (c), and interactions between sward heights and period of evaluation (1 and 2) on herbage mass (b) and bulk density (d). The bars represent the standard errors of the mean.

The total herbage mass and bulk density of the treatments are shown in Fig 1. Herbage mass increased linearly when sward height was assessed in the morning, but in the afternoon, it increased up to 20 cm and then decreased for taller sward heights (Fig 1A), which resulted in a treatment × time-of-day interaction (P < 0.001, S2 Text). The 20 cm treatment resulted in a reduction in herbage mass of approximately 25% from Periods 1 to 2 (Fig 1B), which lead to a treatment × period interaction (P< 0.001, S2 Text). Total herbage bulk density decreased with the sward height, with a significant interaction between treatment and time of day (P < 0.001, S2 Text) and between treatment and period (P < 0.001, S2 Text). The interactions indicated that swards from 15 and 25 cm were denser than other sward heights in the afternoon (Fig 1C), and that 20 cm sward was denser and more abundant in Period 1 than in Period 2 (Fig 1D).

The chemical composition and IVOMD of the hand-plucked forage at different sward heights are shown in Table 2. The NDF values ranged from 48% to 53% of DM, ADF between 17% and 23% of DM, and IVOMD between 58% and 75% of OM (Table 2). The CP values were high and ranging from 27% to 33% (Table 2). The time of day had a strong influence on the fiber content of the collected forage, showing that in the afternoon (PM), swards had lower NDF and ADF than in the morning (Table 2 and S3 Text). Consequently, IVOMD was higher in the afternoon than in the morning, although significant interactions between treatment and time of day and period were observed for IVOMD (P < 0.001, S3 Text). The treatment and time-of-day interaction for IVOMD showed a reduction in taller height mainly in the afternoon assessment, but it was established in Period 2, which also resulted in a significant interaction of treatment by period (P < 0.001, S3 Text). The CP content was lower in the afternoon than in the morning in hand-plucked herbage samples (treatment × time-of-day, P < 0.05, S3 Text). In period 2, the CP content in the 25 cm sward height decreased subtly (treatment × period, P = 0.06, S3 Text). No interactions were found between the time of day and period for any nutritive variable.

**Table 2:** Nutritional value of hand-plucked herbage samples of five sward heights of Kikuyu grass (*Cenchrus clandestinus—*Hochst. ex Chiov) at two times of day in two periods of evaluation.

	Treatment					
	10	15	20	25	30	
	NDF (g/kg of DM)					SEM
Time of day (AM)	519.0	554.1	526.0	539.4	536.0	16.2
Time of day (PM)	495.1	490.1	482.0	498.5	485.0	16.2
Period 1	489.0	516.0	511.5	505.0	525.0	16.2
Period 2	525.5	528.2	496.4	533.1	496.0	16.2
	ADF (g/kg of DM)					SEM
Time of day (AM)	199.4	229.1	210.1	207.5	210.0	9.43
Time of day (PM)	179.2	182.3	181.13	188.0	183.1	9.43
Period 1	179.4	205.0	193.0	194.5	205.2	9.43
Period 2	199.2	206.5	198.2	201.0	188.0	9.43
	CP (g/kg of DM)					SEM
Time of day (AM)	331.0	294.0	316.0	307.0	292.0	11.0
Time of day (PM)	301.4	272.0	284.0	278.0	290.0	11.0
Period 1	318.1	273.0	294.2	296.0	287.0	11.0
Period 2	314.0	293.0	305.13	289.0	295.0	11.0
	IVOMD (g/kg of OM)					SEM
Time of day (AM)	672.2	624.4	588.4	659.1	604.0	12.2
Time of day (PM)	753.2	756.1	714.0	705.4	684.0	12.2
Period 1	733.1	698.2	671.0	706.0	628.1	12.2
Period 2	692.4	682.2	631.4	659.0	660.0	12.2

The times of the day, AM and PM correspond to the morning and afternoon hand-plucked herbage sampling, respectively. SEM: Standard error of the mean.

### Components of ingestive behavior

**Fig 2:**
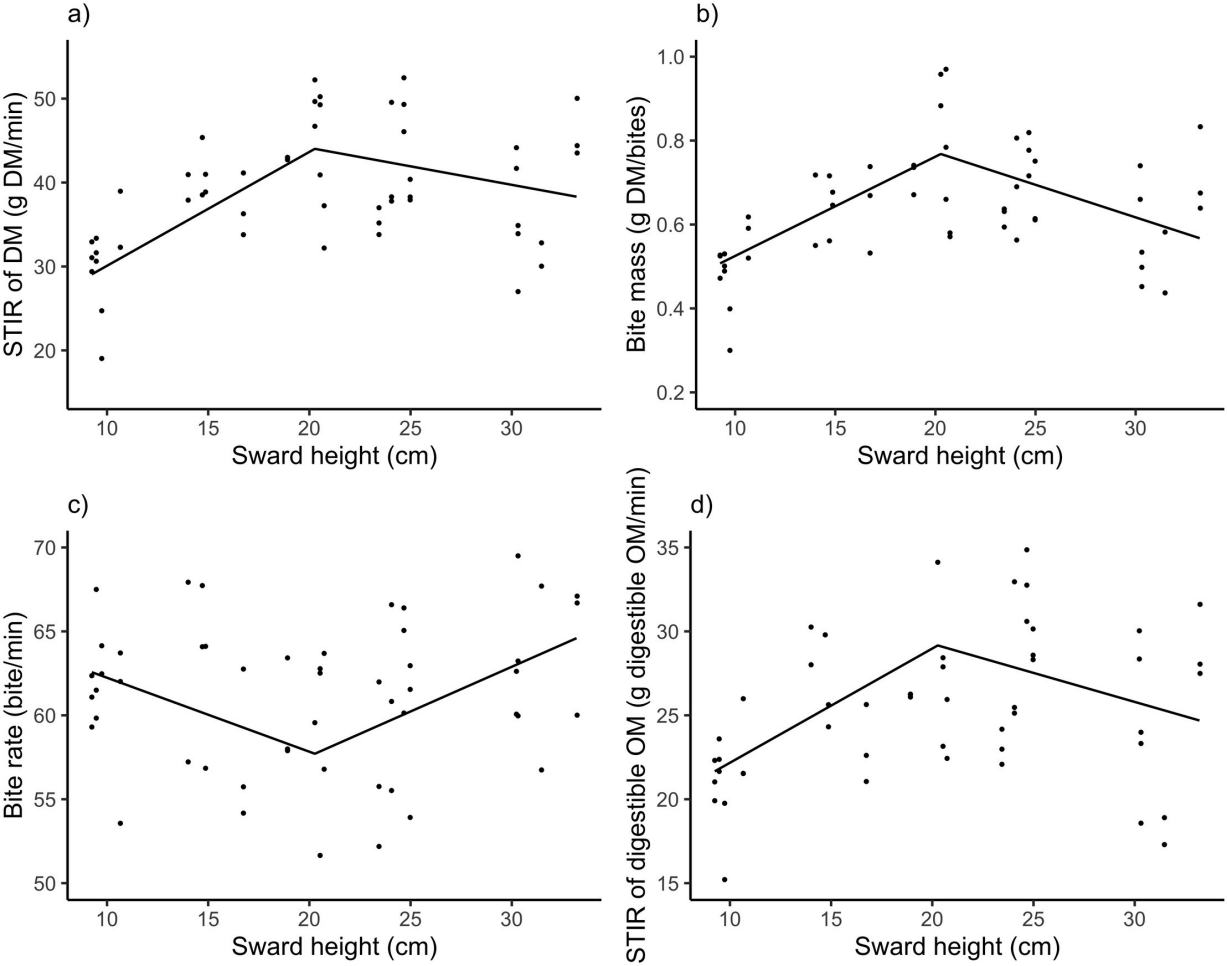
Relationships between ingestive behavior variables and sward height of Kikuyu grass. (a) Relationships between short-term intake rate of the dry matter (STIR of DM), (b) bite mass (BM), (c) bite rate (BR), and (d) short-term intake rate of digestible organic matter (STIR of digestible OM) of dairy heifers as a function of sward height (SH) in monoculture of Kikuyu grass (*Cenchrus clandestinus—*Hochst. ex Chiov). Equation for STIR of DM = min(44 + 1.36 (SH– 19.3), (44–0.44 (SH– 19.3)), P < 0.001, R^2^ = 0.35; BM = min(0.77 + 0.023 (SH– 20.9), (0.77–0.016 (SH– 20.9), P 0.001, R^2^ = 0.36; BR = min(57.71–0.44 (SH– 20.3); (57.71 + 0.53 (SH– 20.3), P < 0.009, R^2^ = 0.14; STIR of digestible OM = min((29.16 + 0.68 * (SH– 20.3); (29.16–0.34 * (SH– 20.3)), P < 0.001, R^2^ = 0.21.

The STIR of the DM model indicated that a maximum STIR of 44 g DM/min was reached with a sward height of 19.3 cm (Fig 2A). The increasing slope (a = 1.36 g DM/min/cm, P < 0.01) was steeper than the decreasing slope (a2 = – 0.44 g DM/min/cm, P < 0.04), (Fig 2A). The BM model achieved a maximum of 0.77 g DM/bite at 20.9 cm of sward height; BM first increased linearly (a1 = 0.023 g DM/cm, P < 0.01) with increasing sward heights up to 20.9 cm, and then decreased (a2 = –0.016 g DM/cm, P < 0.01) (Fig 2B). The minimum BR (57.71 bites/min) occurred at 20.3 cm (Fig 2C). The BR model showed an opposite relationship to BM, first decreasing (a1 = –0.44 bites/min/cm, P = 0.016) up to 20.3 cm, and then increasing (a2 = 0.53 bites/min/cm, P = 0.003) with increasing sward height. The STIR of digestible OM had a similar response to that of DM with a maximum value of 29.16 g of digestible OM/min at 20.3 cm, an increasing slope (a1 = 0.68 g digestible OM/min/cm, P < 0.01) and decreasing slope (a2 = –0.34 g digestible OM/min/cm, P = 0.04), (Fig 2D).

## Discussion

This study showed that an intermediate sward height of approximately 20 cm for Kikuyu grass maximized the STIR of DM and digestible OM by dairy heifers. The intake rate of DM was 30% lower than the maximum in 10 cm swards and 15% lower than the maximum in the 30 cm swards. The BM and STIR constraints in the range below 20 cm are explained by the widely accepted relationships between sward height and bite dimensions (bite area and bite depth) [12,44,45] and the low amount of herbage that the animal can harvest [46,47]. However, for swards greater than 20 cm, these relationships do not explain the decline in the intake rate.

The increase in BR due to the low bite mass [9,11,48] or the reallocation of grazing jaw movements [49,50] was insufficient to maintain the intake rate in the range of short sward heights (<20 cm). Although at taller sward heights (>20 cm), the STIR of DM did not decline as fast as the BM with increasing heights because the increase in BR was steeper than expected based on the increasing side of the response (Fig 2C). This increase was not sufficient to compensate for a small bite mass. We argue that the only possible mechanism for the decline in intake rate is that bite volume declines with increasing sward height, in addition to the observed decline in herbage bulk density.

To elucidate which BM components were involved in the declining phase of the STIR of DM model, we calculated bite dimensions based on mechanistic-empirical models (calculated values, Table 3). Bite volume was calculated as (A) measured bite mass/sward bulk density at the top stratum for each observation averaged over treatments and (B) bite depth × bite area, where bite depth was 0.5 × sward height and bite area was calculated with an empirical model that included the effects of sward height and herbage bulk density of the top stratum [51], and an allometric relationship between dental arcade (DA) breadth and body mass in ruminants [52]. Bite Volume A represents the best approximation of the actual bite volume based on measured quantities, whereas Bite Volume B represents the expected volume based on the generally accepted effects of sward height and density [12,53].

**Table 3:** Foraging behavior variables of dairy heifers grazing Kikuyu grass managed under different sward heights (observed and calculated values).

Variable	Sward height (cm)				
	10	15	20	25	30
	Observed values				
Herbage bulk density of top stratum (g/dm^3^)	0.95	0.68	0.79	0.76	0.61
STIR of DM (g DM/min)	30.6	39.3	44.3	41.3	38.2
BM^A^ (g)	0.50	0.65	0.78	0.68	0.60
BR (bites/min)	61.6	61.2	57.5	60.2	63.4
Bite Volume^A^ (dm^3^)	0.52	0.95	0.99	0.89	0.98
Variable	Calculated values				
Bite Volume^B^ (dm^3^)	0.39	0.71	0.99	1.28	1.71
Bite depth (m)	0.050	0.075	0.10	0.12	0.15
Bite area (dm^2^)	0.78	0.94	0.99	1.02	1.12
BM^B^ (g)	0.35	0.49	0.76	0.95	1.09

STIR of DM, bite mass (BM^A^), and bite rate (BR) are the mean values obtained during the grazing sessions. Bite volume^A^ is the ratio of bite mass to bulk density of top stratum. Bite area was calculated using the equation by [51]bite area = 2*DA*^2^ (1 + 50/SH)^-1^ *e*^(-0.3(*HBd*-1))^, where SH = sward height (cm), HBd = herbage bulk density of the top stratum (g/dm^3^), and DA = dental arcade (cm). DA was calculated using the allometric equation DA = 8.6 BW^0.36^, published by [52], where BW is body weight (kg). Bite volume^B^ was the product of bite area and bite depth. Bite depth was a constant proportion of 50% of sward height. Bite mass (BM^B^) was calculated as the product of bite volume^B^ and bulk density of the top stratum.

Bite Volume A was expected to increase with greater sward height because both bite depth and bite area tended to increase at greater sward heights [12,53], as shown by the changes in Bite Volume B, but this did not occur. In the tall swards in the present experiment, bite volume appeared to remain constant or declined with increasing sward height (Table 3), whereas if it had followed the expected response, bite mass would have increased monotonically with sward height up to 1.09 g; therefore, the decline in bulk density alone cannot explain the observed decline in BM^A^. Thus, the decline in BM and STIR of DM for swards taller than 20 cm can be explained by both a decline in bulk density and an unexpected decline, or the lack of the usually observed increase in bite volume.

Bite mass and STIR constraints by the sward structure, especially sward height and bulk density, are well recognized in research at the plant-animal interface [12,54,55]. However, reductions in bite volume at greater sward heights are less common. Some authors argue that bite mass and STIR reductions at high sward heights are due to increments in time per bite associated with decreasing bulk density in the upper stratum of the sward [10,18,56]. Similarly, it is widely recognized that the presence of stems + sheaths in the grazed stratum constrains the bite mass and herbage intake rate [45,47,57]. According to [17], the BM decreases in tall sward heights of *Cynodon sp* (cv. Tifton 85) because of the increasing proportion of less desirable plant parts, such as stems and sheaths, which reduces the bite volume. In this study, the stems + sheath mass had a similar response to the total herbage mass, increasing with sward height and showing an interaction between sward height and time of day and period of evaluation. Previous studies on three-dimensional sward structures at the bite scale have shown a greater dispersion of leaf blades in the upper stratum with increasing sward height in tall pastures [58–60]. We hypothesize that the influence of the stem and sheath mass and the declines in total bulk density and leaf bulk density with sward height resulted in changes in the spatial distribution of the leaves in the top stratum, which resulted in reductions in the bite volume, and thus in smaller bites in tall swards.

The time of day for hand-plucked herbage had a marked effect on NDF and ADF. In contrast, the interaction between sward height, time of day, and evaluation period influenced both CP and IVOMD. Variations in herbage chemical composition due to time of day, grazed stratum, evaluation period, or interactions have previously been described and discussed in the literature [20,61,62]. Our results concerning the NDF, ADF, CP, and IVOMD were consistent with the values found in the upper stratum of Kikuyu sward [20,63]. However, CP exhibited higher values than those usually reported for the grazing layer of Kikuyu grass pastures [22,64].

In the morning, the swards had higher CP content, lower IVDMD, and higher NDF and ADF than those in the afternoon. Studies on the vertical distribution of biomass and chemical composition have suggested that concentrations of photosynthates in leaves have greater diurnal fluctuations than that of stems and pseudostems; therefore, NDF and CP concentrations may be diluted in the DM as the day progresses [61,65,66]. Consistently, other studies have shown that the CP content of leaves changes significantly with the stage of regrowth of the pasture [67] and even with anatomical characteristics along the length of the leaf blades [68]. The high CP values can also be explained by the higher N content in leaf blades than in stems and sheaths due to the N fertilization [69].

Even considering the fluctuation of time of day and period in herbage chemical composition, as the grazed stratum contained mainly highly digestible leaves with similar nutrient content between sward heights, the maximization of the STIR of DM and digestible OM occurred concomitantly in this study. The similarities in nutritive value between sward heights can be attributed to the fact that all pastures were still in their vegetative stage. For a given stratum of the sward, the differences between regrowth ages were commonly more marked between vegetative and reproductive stages [20,64]. In the vegetative stage, the nutritive value differs little among the plant parts [20,58]. Although additional work is required to fully understand the differences and relationships between the intake rate of DM and nutrients, our results concerning the intake rate of nutrients are consistent with findings reported in the scientific literature in which DM intake rate maximization has been incorporated into the maximization of nutrient intake rate [14,16–18]. Finally, if grazed herbage is to be an important source of nutrients for a dairy cow to meet its nutritional requirements, it is reasonable to set grazing management targets that provide the longest pasture access time of Kikuyu grass of 20 cm.

## Conclusions

To maximize the short-term intake rate (STIR) of DM and digestible OM of dairy heifers under rotational stocking, the pre–grazing sward height of the Kikuyu grass should be managed at 20 cm. Very low (10 cm) or high (30 cm) sward heights of Kikuyu grass as grazing management targets would constraint the BM, and thus, the STIR.

## Supporting information

S1 Text Summary of Anova results for the fitted linear mixed-effects of pre- and post-grazing sward heights of Kikuyu grass. (TXT)

S2 Text Summary of Anova results for the fitted linear mixed-effects of sward structure characteristics of Kikuyu grass. (TXT)

S3 Text Summary of Anova results for the fitted linear mixed-effects of Nutritional value of hand-plucked herbage samples of Kikuyu grass. (TXT)
